# Supplementary material for: Nucleosome-bound NR5A2 structure reveals pioneer factor mechanism by DNA minor groove anchor competition
Source: Nat Struct Mol Biol. 2024 Feb 26;31(5):757–66. doi: 10.1038/s41594-024-01239-0 (PMC11102866; doi:10.1038/s41594-024-01239-0)

For Figure 5e

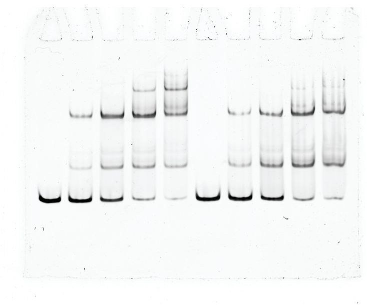

For Figure 5f, SYBR safe detection

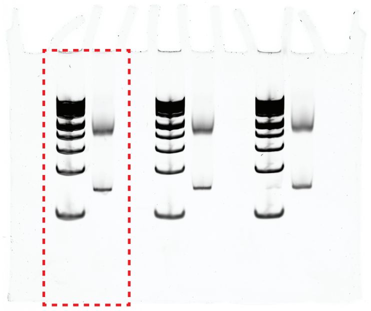

For Figure 5f, Alexa 647 detection

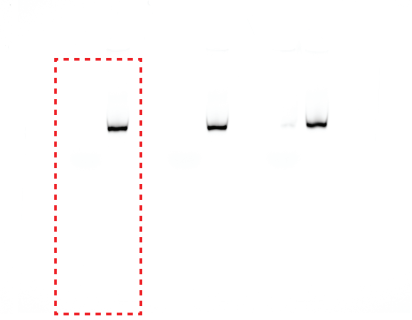

For Figure 5g, SYBR gold detection

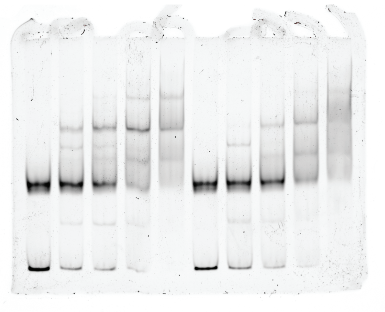

For Figure 5g, Alexa 647 detection

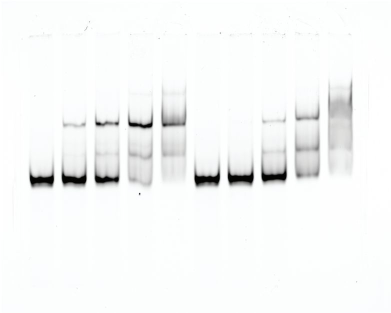

Supplement: Supplementary file 8 — Unprocessed gels [file 41594_2024_1239_MOESM8_ESM.pdf]
