# Supplementary material for: Nucleosome-bound NR5A2 structure reveals pioneer factor mechanism by DNA minor groove anchor competition
Source: Nat Struct Mol Biol. 2024 Feb 26;31(5):757–66. doi: 10.1038/s41594-024-01239-0 (PMC11102866; doi:10.1038/s41594-024-01239-0)

For Extended Data Figure 7b,
Alexa647 detection

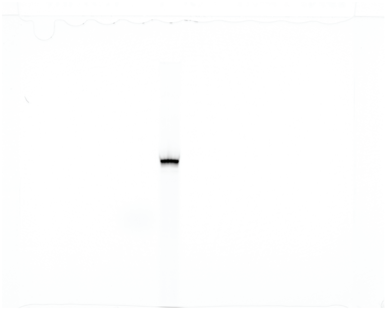

For Extended Data Figure 7b,
Cy3 detection

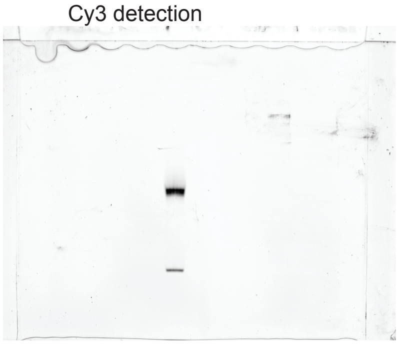

For Extended Data Figure 7b,
SYBR safe detection

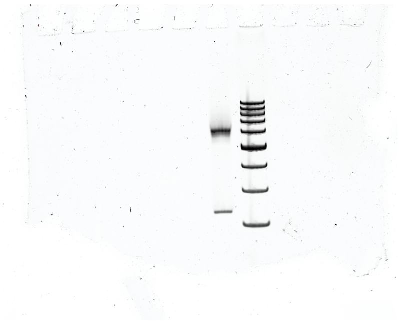

For Extended Data Figure 7c,
Alexa647 detection

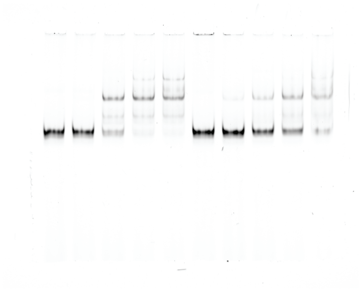

For Extended Data Figure 7c,
Cy3 detection

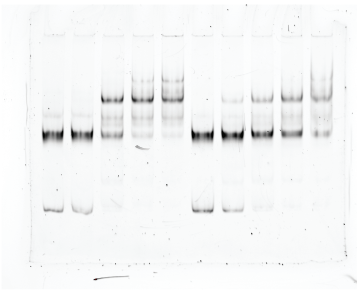

Supplement: Supplementary file 13 — Unprocessed gels [file 41594_2024_1239_MOESM13_ESM.pdf]
