# Supplementary material for: Nucleosome-bound NR5A2 structure reveals pioneer factor mechanism by DNA minor groove anchor competition
Source: Nat Struct Mol Biol. 2024 Feb 26;31(5):757–66. doi: 10.1038/s41594-024-01239-0 (PMC11102866; doi:10.1038/s41594-024-01239-0)

For Extended Data Figure 8b

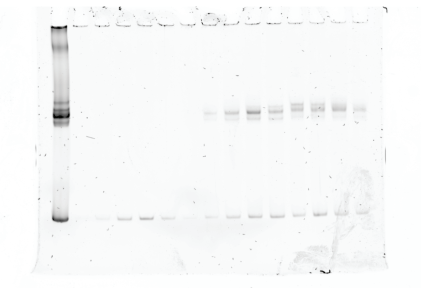

For Extended Data Figure 8c

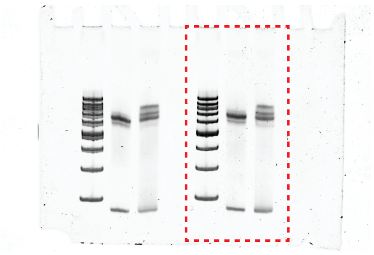

For Extended Data Figure 8e

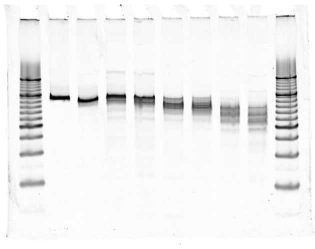

For Extended Data Figure 8g

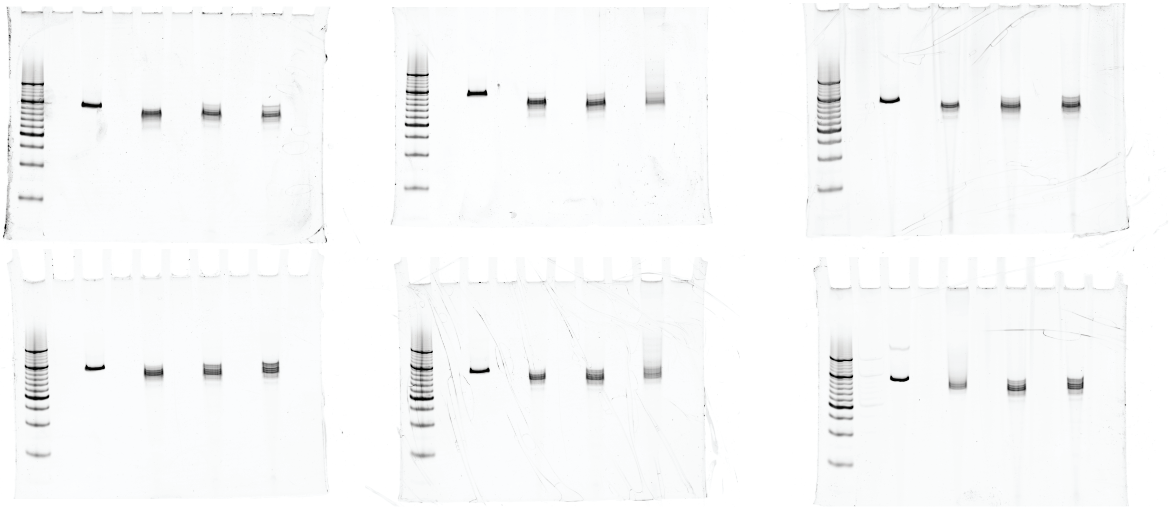

For Extended Data Figure 8i

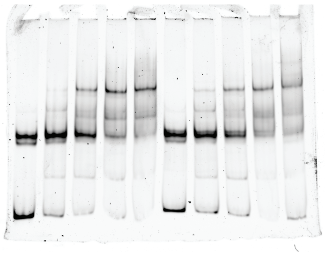

Supplement: Supplementary file 15 — Unprocessed gels [file 41594_2024_1239_MOESM15_ESM.pdf]
